# Early detection of osteoarthritis in the rat with an antibody specific to type II collagen modified by reactive oxygen species

**DOI:** 10.1186/s13075-021-02502-1

**Published:** 2021-04-14

**Authors:** Anne Gigout, Donata Harazin, Louise M. Topping, Didier Merciris, Sven Lindemann, Christian Brenneis, Ahuva Nissim

**Affiliations:** 1 Osteoarthritis Research, Merck KGaA, Darmstadt, Germany; 2 Barts and the London School of Medicine and Dentistry, Queen Mary University of London, Chaterhouse Square, London, EC1M 6BQ UK; 3 Galapagos SASU, Romainville, France

**Keywords:** Reactive oxygen species, Osteoarthritis, Hypertrophy, Collagen type II, Collagen type X

## Abstract

**Background:**

Osteoarthritis (OA) is a disease of the whole joint, with articular cartilage breakdown as a major characteristic. Inflammatory mediators, proteases, and oxidants produced by chondrocytes are known to be responsible for driving cartilage degradation. Nevertheless, the early pathogenic events are still unclear. To investigate this, we employed an antibody that is specific to oxidative post-translationally modified collagen type II (anti-oxPTM-CII) to detect early cartilage pathogenic changes in two rat models of OA.

**Methods:**

The animals underwent surgery for destabilization of the medial meniscus (DMM) and were sacrificed after 3, 5, 7, 14, and 28 days. Alternatively, anterior cruciate ligament transection with partial meniscectomy (ACLT+pMx) was performed and animals were sacrificed after 1, 3, 5, 7, and 14 days. Joints were stained with toluidine blue and saffron du Gatinais for histological scoring, anti-oxPTM-CII, and anti-collagen type X antibodies (anti-CX).

**Results:**

We observed positive oxPTM-CII staining as early as 1 or 3 days after ACLT+pMx or DMM surgeries, respectively, before overt cartilage lesions were visible. oxPTM-CII was located mostly in the deep zone of the medial tibial cartilage, in the pericellular and territorial matrix of hypertrophic chondrocytes, and co-localized with CX staining. Staining was weak or absent for the lateral compartment or the contralateral knees except at later time points.

**Conclusion:**

The results demonstrate that oxidant production and chondrocyte hypertrophy occur very early in the onset of OA, possibly initiating the pathogenic events of OA. We propose to use anti-oxPTM-CII as an early biomarker for OA ahead of radiographic changes.

## Background

Osteoarthritis (OA) is one of the leading causes of reduced quality of life worldwide, due to the associated chronic pain and various degrees of disability. Although OA affects all the tissues of the articular joint, degradation and loss of articular cartilage is a central feature [1, 2]. Cartilage degradation in OA results from a disruption in homeostasis due to activation of the chondrocytes by various factors that promote the production of matrix degrading enzymes, in excess of the capacity of the chondrocyte to replace damaged and degraded matrix components. The factors that activate chondrocytes to promote matrix degradation include excessive and abnormal mechanical loading, pro-inflammatory cytokines, and chemokines, as well as Wnt ligands and factors activating the innate immune system [1, 3]. Many of these OA factors stimulate chondrocytes to produce reactive oxidants (ROS). ROS are utilized as secondary messengers in mediating intracellular signaling events that regulate expression of matrix degrading enzymes [4, 5] and pro-death signaling pathways, thus compromise chondrocyte integrity and promote cartilage damage [6]. The most abundant ROS produced by chondrocytes include superoxide, hydrogen peroxide, the reactive nitrogen species nitric oxide, and the nitric oxide derived product peroxynitrite [2].

Experimental OA models induced by joint instability have been highly valuable in identifying key pathogenic pathways in disease and for validating new treatments. They produce robust degradation of the articular cartilage and changes in the subchondral bone and can be used to investigate pain-like symptoms. Widely used models of animal OA involve surgically induced instability of the knee [7]. These models are characterized by an acute injury to the joint that causes mechanical instability, resulting in OA. Many types of operations on various animals have been developed, including cruciate or collateral ligament transections and partial or total meniscectomies on dogs, goats, rabbits, and rodents. Most of the studies detect mild changes in the articular cartilage at 2 to 4 weeks post-operatively. For example, in the anterior cruciate ligament transection (ACLT) model, cartilage destruction is seen 2–4 weeks after surgery [8]. Alternatively, when OA is induced by destabilization of the medial meniscus (DMM), structural-change progression is slower [9–11]. Currently, the size of the animal precludes prospective assessment of disease by conventional radiographic approaches, and disease is assessed by serial histology of the joint, which is time consuming, costly, and requires large number of animals as they need to be culled at each experimental time point under investigation. Powerful non-invasive small-animal imaging techniques for longitudinal studies are therefore highly desirable for preclinical validation studies as well as for detection and monitoring of early OA in patients.

We previously developed a panel of human single chain fragment variable (scFv) that binds specifically to oxidative post-translationally modified collagen type II (oxPTM-CII) [12]. We showed that anti-oxPTM-CII (i) binds specifically to arthritic cartilage from patients with RA and OA; (ii) stains cartilage in murine models of inflammatory arthritis (antigen induced arthritis (AIA) and OA, namely DMM); (iii) localizes in the arthritic joint in vivo in a mouse model of AIA and DMM following systemic administration of labeled anti-oxPTM-CII with Alexa Fluor 680 or Cy5.5 [12, 13]; and (iv) was able to target therapeutic scaffolds specifically to arthritic joint [14].

In the current study, we evaluate longitudinally the presence of oxPTM-CII staining in early OA in two rat models: DMM and ACTL+pMx. Our goal was to possibly unveil some of the very early events in OA and evaluate the possibility to detect the initiation of the disease before the appearance of cartilage lesions. Rats were sacrificed 3, 5, 7, 14, and 28 days after DMM and 1, 3, 5, 7, and 14 days after ACTL+pMx surgery and the lateral and contralateral knees were stained with toluidine blue and saffron du Gatinais, or for oxPTM-CII and type X collagen (CX). OxPTM-CII and CX were detectable already at the earliest time points in the medial tibial cartilage and strongly co-localized together. We conclude that (i) ROS production and increased type X collagen expression are early events in OA and prefigure cartilage lesions and (ii) anti-oxPTM-CII detection could be a powerful tool to detect initiation of OA.

## Method

### Antibody preparation

Anti-oxPTM-CII scFv was expressed in HB2151 bacteria as described [15]. ScFv was converted to full length antibody by cloning V_H_ domain into pFUSEss-CHIg-hG1e3 and V_L_ domain into pFUSEss-CLIg-hk (InvivoGen). Plasmid DNA was isolated using a QIAFilter Plasmid Maxi Kit according to the manufacturer’s instruction (QIAGEN). Following transient expression in Expi293F Expression System according to the manufacturer’s instructions (Thermo Fisher Scientific), supernatants were collected and purified using protein A Sepharose CL-4B (GE Healthcare). The ability to retain specific binding of anti-oxPTM-CII over native CII was assessed by ELISA as described [12].

### Animal models

Male Lister Hooded (Crl:LIS) outbred SPF rats (aged 8–9 weeks and within 150–175 g weight range, Charles River) were housed in colony cages as described [16] with 48 rats/cage at the start of the study. After 4 weeks of acclimatization, rats underwent surgery under anesthesia. Anterior cruciate ligament transection with partial meniscectomy (ACLT+pMx) was performed as described elsewhere [16] with the exception that only 50% of the meniscus was removed. For destabilization of the medial meniscus (DMM), a skin incision was made from distal the patella proximal to the tibial plateau (of the right joint). The muscle layer was opened in knee flexion with a scalpel and prepared to visualize the medial meniscus tendon which was ligated using scissors. Finally, the joint capsule, associated muscles and connective tissue were sutured in layers. For postsurgical analgesia, rats received meloxicam (0.5 mg/kg s.c.; Metacam injection solution, Boehringer Ingelheim). At the specified time points following surgery, rats (*N* = 9–10 per time point) were humanely euthanized by transthoracic heart puncture under isoflurane anaesthetization.

### Histology and scoring

The ipsilateral knees of all animals and contralateral knees of two animals per time points (*N* = 10) were fixed for 7 days in paraformaldehyde (VWR) 4% in phosphate-buffered saline (PBS, VWR) and decalcified for 6 weeks in formic acid (Sigma-Aldrich) 4% in PBS and embedded in paraffin. Coronal sections of 7 μm (including medial tibial plateau, femur condyle, and menisci) were cut with a microtome within the weight-bearing area. Every 35th section was collected. The sections were deparaffinized and rehydrated, stained with toluidine blue (VWR, 0.05% in PBS, 6 min) and saffron du Gatinais (Morphisto GmbH, diluted 1:3 in absolute Ethanol, 1 min). Slides were then dehydrated, coverslipped, and after drying digitized using slide scanner SCN400 (Leica Microsystems). For histopathological grading, we adapted the modified Mankin score described in [17] and recommended by the OsteoArthritis Research Society (OARSI) for guinea pig. The changes we made to this score had the purpose to make it usable for several species, to be able to compare results obtained with various types of animals. In the present work, we used it to describe rat OA. Our score has a maximum SUM score of 28. Sub-scores are described in supplementary material Table S1. Scanned sections were analyzed by two independent observers and the most severe lesion within the weight-bearing area for each rat was selected for evaluation. For each sub-score, the values from two consecutive sections were averaged to determine overall values for each animal.

### Immunostaining

Single or double staining for type X collagen (CX) and ROS-modified type II collagen (oxPTM-CII) were applied. For the ACLT+pMx study, two single staining were conducted, one for CX and one for oxPTM-CII. For the DMM study, to evaluate the co-localization of CX and oxPTM-CII a double staining was first performed. However, because the blue staining of CX was very dark and both staining overlapped, it was difficult to visualize clearly the brown staining of the oxPTM-CII. For this reason, for the DMM study, an additional single staining for oxPTM-CII was conducted.

For the CX staining, the sections were first deparaffinized and rehydrated. An epitope retrieval using Proteinase K (Leica Biosystems) [18] was performed. Sections were subsequently incubated with a monoclonal mouse antibody specific for Collagen X (#1-CO097-05, Quartett) diluted 1:50, for 30 min at room temperature. For the detection, the Leica Polymer Refine Red Detection System (#DS9390, Leica Biosystems) was used where the RED dye was substituted with NBT/BCIP (#ab7468, Abcam). The type X collagen staining was developed using a fully automated immunohistochemistry stainer (Bond III, Leica Microsystems).

For the staining of oxPTM-CII, epitope retrieval was performed with pepsin. Sections were first equilibrated in HCl (Merck KGaA) 0.02% in ddH_2_O, 37 °C, 15 min and digested with 15 mg/mL pepsin (Merck KGaA) in HCl 0.02%, 37 °C, 45 min [19]. The sections were incubated with the anti-oxPTM-CII antibody 6.5 μg/mL overnight at 4 °C and detected using Polink-2 Plus HRP human IgG with 3,3′-diaminobenzidin (DAB) (#D88, GBI Labs).

For both staining protocols, negative controls were performed where the primary antibody was omitted.

CX and oxPTM-CII staining were quantified in a region of interest (ROI) selected on the tibial medial plateau using the image analysis software, Calopix® v 4.1.0.3 (Tribvn, France). Positive areas for each staining were expressed as the percentage of the total cartilage area in the ROI. The ROI was placed where lesions develop and was 1 mm long (the width corresponded to cartilage thickness). Because the growth plate was found to be positive for both oxPTM-CII and CX, sections that were not or only weakly stained in this area were excluded from the analysis.

### Statistical analysis

Data were analyzed for their normality with the D’Agostino Person or the Shapiro-Wilk normality test (for *n* < 8). For most of the data, several groups did not follow a normal distribution and the Kruskal-Wallis test corrected for multiple comparison with a Dunn’s test was applied. For the oxPTM-CII quantification results in the DMM and ACLT+pMx model and the CX quantification in the ACLT model, all groups followed a normal distribution and in this case, a one-way ANOVA corrected for multiple comparison with a Dunnet test was applied. GraphPad Prism v8.4.2 was used.

## Results

### DMM surgery affects more profoundly and ACLT+pMx more strongly the medial tibial compartment

**Fig. 1:**
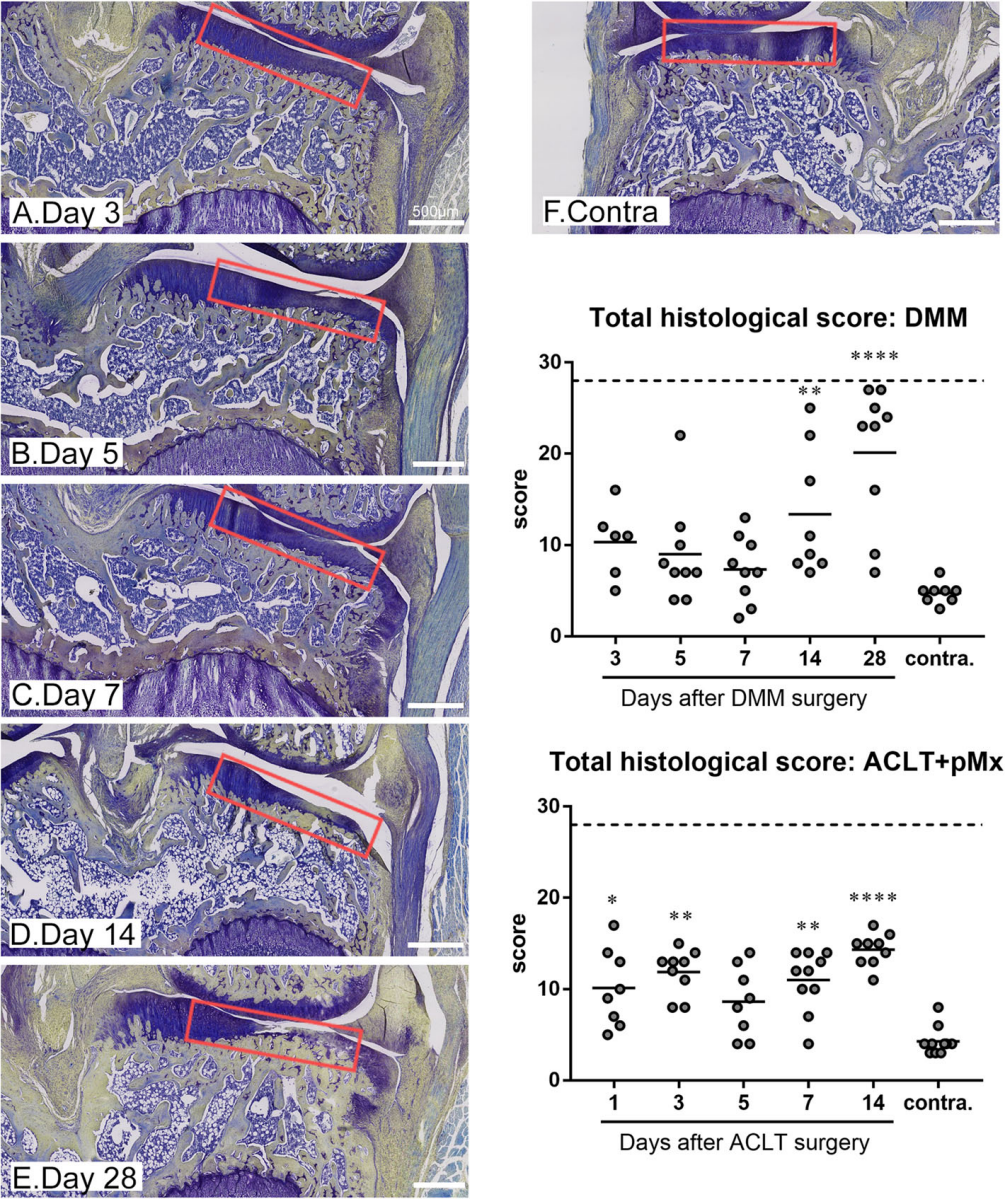
Histological scoring of OA for the medial tibial plateau in the ACLT+pMx and DMM models. Rats underwent ACLT+pMx or DMM surgery and were sacrificed at various time points (*N* = 9–10 rats per time points). The ipsilateral or contralateral knees were taken for histological analysis. Slides were stained with toluidine blue and saffron du Gatinais and scored as detailed in the “Method”. Histological sections for the DMM model are shown with the region selected for scoring marked in red. The total histological score is shown for both models. Data on the graphs represent the total score for each animal (*N* = 9–10) and the mean for each time point and for the selected contralateral knees. Double asterisks and quadruple asterisks mean significantly different from contralateral with *p* < 0.01 or *p* < 0.0001, respectively

After both DMM and the ACLT+pMx surgeries, osteoarthritis develops mostly in the medial tibial plateau and this area was scored according to the histochemical-histological scoring system modified from [17]. For the DMM model, animals were sacrificed at days 3, 5, 7, 14, and 28. Because ACLT with meniscectomy results in more severe OA compared to DMM [9], earlier time points were chosen for this model and animals were sacrificed at days 1, 3, 5, 7, and 14. After DMM surgery, loss of matrix staining in cartilage was apparent already after 3 days as illustrated on Fig. 1a and on the matrix staining sub-score (Figure S1) but cartilage defects appeared only at day 28 in most of the animals (Fig. 1e and sub-score cartilage structure in Figure S1). Most of the other sub-scores such as cellularity, alteration of the tidemark and thickening of the subchondral bone started to be evident at day 14. Small to medium-sized osteophytes were observed in a minority of animals 14 or 28 days after the surgery. As a result, the total histological score was significantly elevated at day 14 and 28 in comparison to the contralateral medial tibial plateau (Fig. 1) for this model. After the ACLT+pMx surgery, the total histological score (Fig. 1) was significantly elevated from the first day compared to the contralateral knee and this was mainly driven by the cartilage structure and the matrix staining sub-scores (Figure S2). The other sub-scores were barely affected, and no osteophyte was observed. The comparison of both models at days 3–14 shows that the total histological scores were higher (except at day 5) for the ACLT+pMx model, and this seems to be mostly driven by the higher sub-scores for cartilage structure and matrix staining. However, more sub-scores were affected in the DMM compared to ACLT+pMx.

### OxPTM-CII and CX signal in the deep zone are early markers of OA

**Fig. 2:**
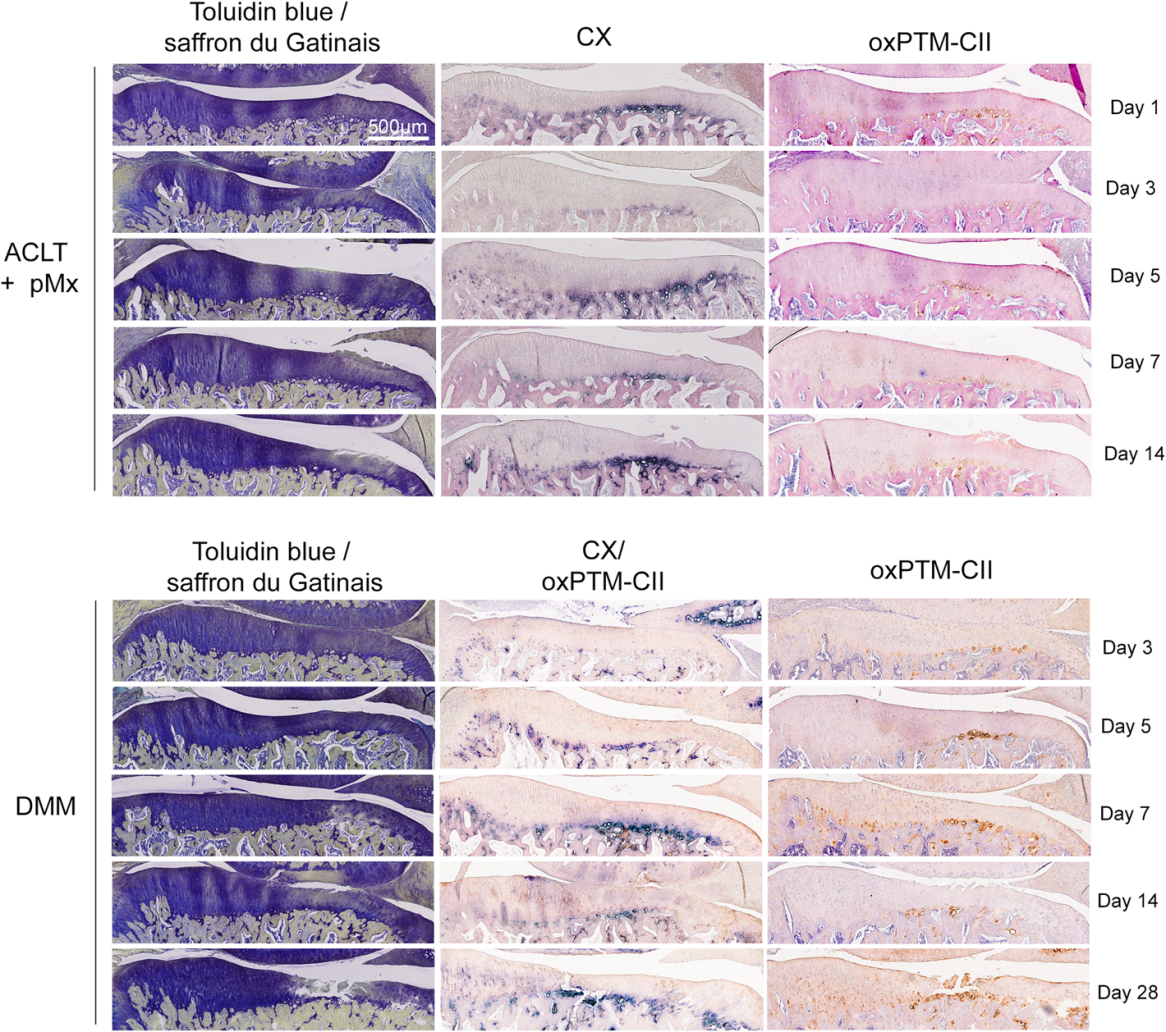
CX and oxPTM-CII staining of the medial tibial plateau cartilage in the ACLT+pMx and DMM models. Representative results for toluidine blue and saffron Gatinais as well as type X collagen (blue, CX) and oxPTM type II collagen (brown, oxPTM-CII) immunostainings are shown. For the ACLT+pMx, study single immunostainings were realized. For the DMM study, a CX and oxPTM-CII double staining was performed as well as an oxPTM-CII collagen single staining

ACLT+pMx and DMM knees were stained with anti-oxPTM-CII and antibody specific to CX (staining for the medial tibial plateau is shown in Fig. 2 and for the lateral tibial plateau in Figure S3). On the medial compartment (Fig. 2), for both the ACLT+pMx and DMM knees, a strong CX staining is observable in the deep zone located where matrix loss or cartilage damages were also visible. Otherwise, in the rest of the deep zone, the staining was weak all along the tidemark. Similarly, most of the cartilage was negative for oxPTM-CII but a positive staining was evident in the deep zone in the region where cartilage degradation occurred. Interestingly, both staining were already observable at early time points, before visible cartilage damage started to develop. Both staining were also particularly strong where hypertrophic chondrocytes in the deep zone were visible. In addition, in the DMM model at day 28, more severe damage could be observed. In this case, oxPTM-CII and CX staining extended beyond the deep zone at the damage site and the full depth of cartilage was positive for CX.

We also looked at the lateral tibial compartment of the operated knees (Figure S3) and the contralateral knees (Figure S4). Both the ACLT+pMx and DMM models showed weak or no visible staining in the lateral tibial compartment at early time points (days 1 or 7 or days 3 or 14, respectively) for CX and oxPTM-CII irrespective of the histological score obtained on the medial side (indicated in brackets Figure S3A). However, at day 14 in the ACLT+pMx model and day 28 in the DMM model signal for both collagens appeared. Contrary to the medial tibial cartilage, the staining was not restricted to a specific location in the cartilage. These results illustrate that at later time points the disease progressed to the lateral side of the operated joint. The lateral tibial compartment was scored as well (Figure S3B) and the histological scores were low in all groups and no difference could be observed to the contralateral lateral tibial compartment at all time point tested.

Similarly, the contralateral knees (Figure S4) showed only a weak CX and oxPTM-CII staining at day 1 or 3 in the ACLT+pMx or DMM models respectively but an increased staining intensity was visible on the medial side for both CX and oxPTM-CII after ACLT+pMx at day 14 and for oxPTM-CII after DMM at day 28. As for the ipsilateral knee, CX and oxPTM-CII were strictly localized in the deep zone around the large hypertrophic chondrocytes in the medial tibial compartment, and the staining appeared more diffuse in the lateral tibial compartment.

**Fig. 3:**
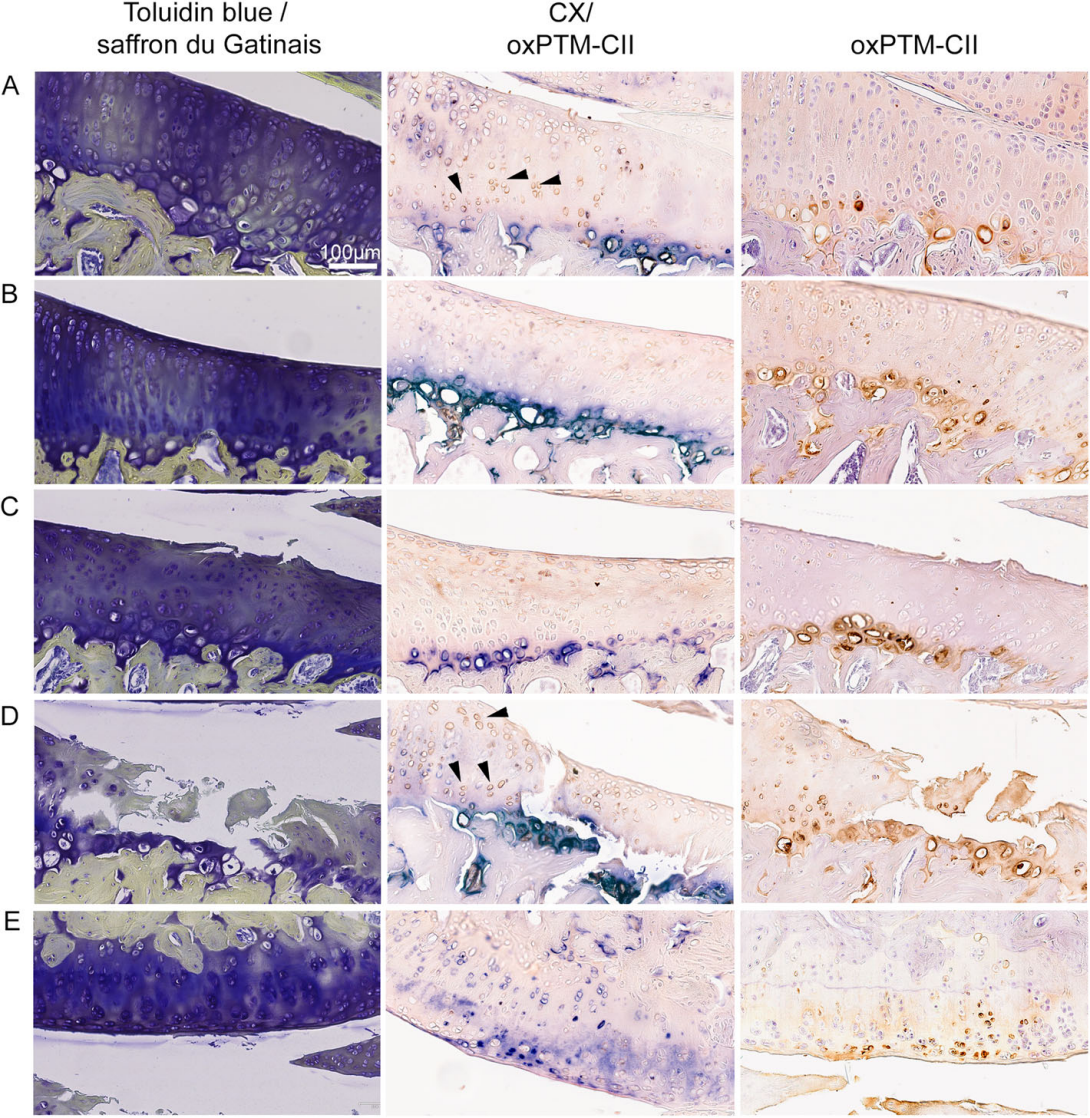
Partial co-localization of CX and oxPTM-CII staining in the cartilage of the medial tibial plateau and condyle in the DMM model. Toluidin blue and saffron du Gatinais, type X collagen (blue, CX) and oxPTM type II collagen (brown, oxPTM-CII) staining are shown for cartilage of the medial tibial plateau presenting different levels of degeneration: **a**, **b** loss of matrix staining but no defect (both from day 14) and **c**, **d** cartilage with a small and a large defect, respectively (from day 5 and 28, respectively). One example of staining for cartilage form the medial condyle is shown in **e**. Black arrows show examples of chondrocytes positive for oxPTM-CII but negative for CX

Finally, condylar cartilage was found to be mostly negative for oxPTM-CII (not shown) but started to be positive when large damages develop on the tibial side (see example on Fig. 3d, e). Condylar cartilage was also weakly positive for CX, which was strictly localized in the deep zone. A stronger CX staining appeared with the progression of the disease but at later time points compared to the tibial cartilage (data not shown).

### OxPTM-CII and CX staining partially co-localize

Higher magnifications of the medial tibial cartilage from DMM knees are shown in Fig. 3 for cartilage with matrix staining loss but no defect (Fig. 3a, b) or for cartilage with a small (Fig. 3c) or a large defect (Fig. 3d). An example for condylar cartilage is also shown (Fig. 3e). Only the DMM knees are shown as the double staining for CX and oxPTM-CII enabled to better determine whether both markers co-localized. In the cartilage with no apparent defect, CX and oxPTM-CII staining co-localized in the deep zone mostly in the pericellular and territorial matrix of the hypertrophic chondrocytes. In panel A, chondrocytes that were positive for oxPTM-CII but negative for CX can also be observed (see arrows). In cartilage presenting a small defect (panel C), the pattern was similar. However, in the case of larger defect (panel D), CX staining was found in the interterritorial matrix and extended to the middle zone while oxPTM-CII staining extended to the middle and superficial zone. Interterritorial staining of oxPTM-CII was also observed in the middle and superficial zones in the area of the damaged fibrillar cartilage (panel D). In addition, in the middle and superficial zones, cells positive for oxPTM-CII but no CX were observed (see arrows). These results indicate that oxPTM-CII and CX staining mainly co-localize (around the larger chondrocytes in the deep zone) in early OA but might show a slightly different pattern at later OA stages. Finally, condylar cartilage facing a large defect (here picture from panels D and E are from the same animal) were positive for both staining and the staining were observed in all zones of cartilage.

### The area of the cartilage positive for oxPTM-CII and CX increases with the progression of the disease

**Fig. 4:**
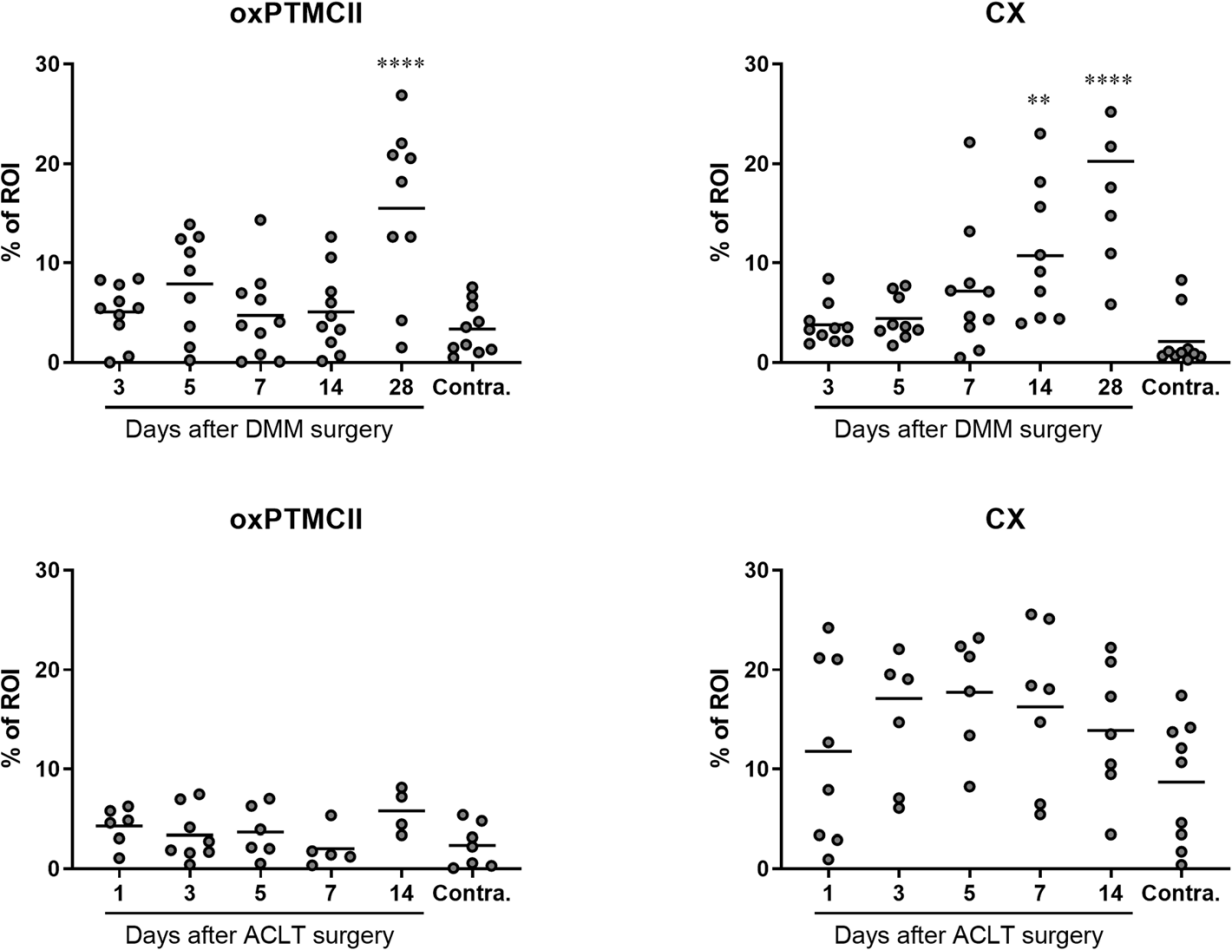
Quantification of oxPTM-CII and CX stainings in the cartilage of the medial tibial plateau in the DMM and ACLT+tMx models. A region of interest (ROI) was defined in the medial tibial plateau and the area stained in blue (for CX) or in brown (for oxPTM-CII) was measured and normalized by the total cartilage area in the ROI. Data on the graphs represent the % area of the ROI for each animal (*N* = 4–10) and the mean for each time point and for the selected contralateral knees. A single asterisk, double asterisks, and quadruple asterisks mean significantly different from contralateral with *p* < 0.05, 0.01, or 0.0001, respectively

The areas positively stained for oxPTM-CII and CX were quantified in the medial tibial cartilage for both models (Fig. 4). In the DMM model, the oxPTM-CII and CX positive areas were larger in the operated knee compared to the contralateral knee and their size increased with disease progression. Compared to contralateral knees, this increase was not significant at early time points and became significant at day 28 for oxPTM-CII and days 14 and 28 for CX. These results strengthen the previous observations that oxPTM-CII and CX extend progressively beyond the deep zone as OA progresses.

 For the ACLT+pMx model, the stained area for oxPTM-CII remained small, and similarly to the DMM model no difference to the contralateral medial cartilage could be observed at day 14 or before. However, a trend to increased oxPTM-CII can be observed at day 14 (*p* = 0.0920). The CX staining was found to be highly variable. There was a trend to an increased positive area at day 3 to 14 (only significant at day 5) but no clear progression can be observed. These results are in accordance with the histological score (Fig. 1) which did not progress for the ACLT+pMx study between day 1 and 14.

Finally, it would have been interesting to compare the operated knees to healthy rat knees instead of collateral knee of OA rats, to evaluate if a significant difference for oxPTM-CII and CX can appear at earlier time points. Indeed, in this study, the contralateral knees were selected from all time points (2 rats per time point) and as we described above, the positive areas for oxPTM-CII and CX started to increase contralateral knees at day 14 for the ACLT+pMx and day 28 for the DMM models. This would suggest that for this readout contralateral knees are not equivalent to those of healthy controls.

### The growth plate and the meniscus are positive for oxPTM-CII

**Fig. 5:**
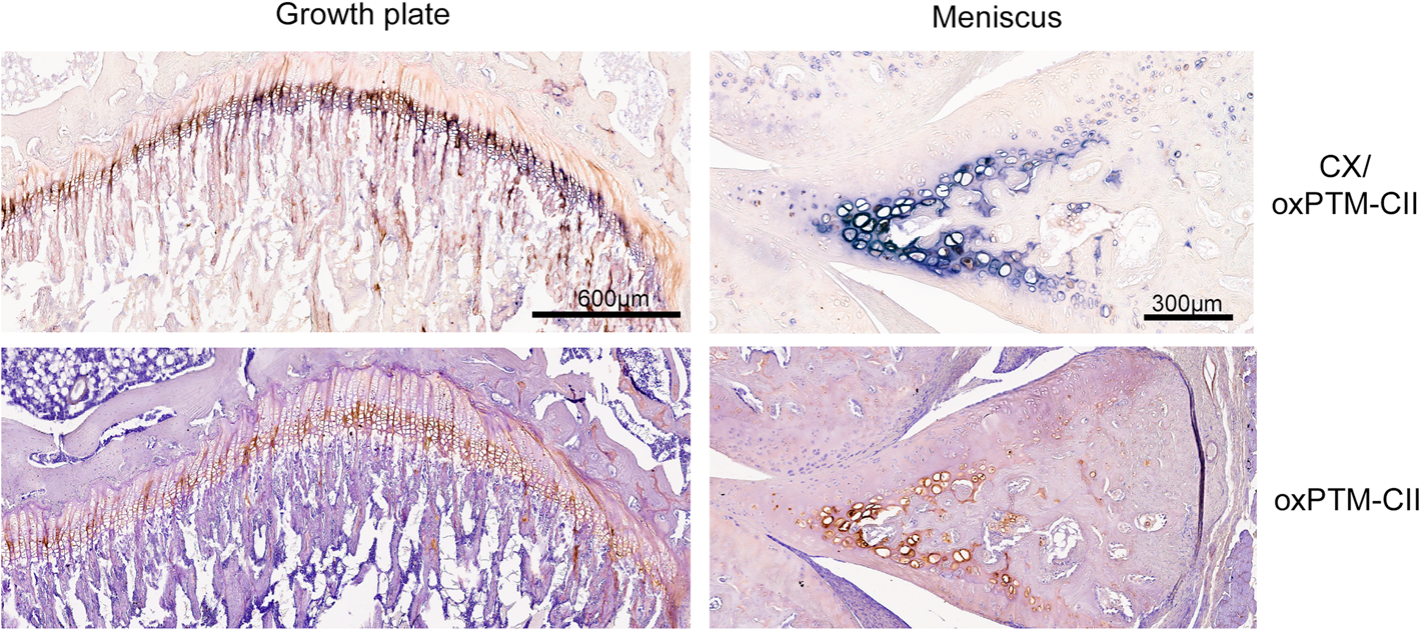
CX and oxPTM-CII staining in the meniscus and in the growth plate. One example of a double staining for type X collagen (blue, CX) and oxPTM type II collagen (brown, oxPTM-CII) and the single staining for oxPTM-CII is shown for the growth plate and the meniscus

As expected, the growth plate and the calcified part of the meniscus were positive for CX (Fig. 5). These tissues were also found to be positive for oxPTM-CII. In these two tissues, both staining appear to co-localize.

## Discussion

The ACLT+pMx (or tMx) and DMM models are both surgically induced instability OA models that are broadly used to study disease progression. In the present study, we investigated early disease progression for both models and evaluated the presence of oxidized type II collagen in cartilage and its co-localization with type X collagen—a marker of chondrocyte hypertrophy.

In the current study, we used an ACLT+pMx and DMM model in rats housed in colony cages enabling free movement of the animals [9, 16]. In accordance with previous results from others [9], the total histopathological scores were slightly higher with the ACLT+pMx model than the DMM model for the same time points and the diseased developed earlier with the ACLT+pMx. Prolonging both studies for a longer time could have enabled to observe bigger difference between both models [20]. Interestingly, when looking at the sub-scores, the ACLT+pMx influenced only two categories (cartilage structure and matrix staining) until day 14, whereas after DMM all sub-scores were affected.

Oxidative stress is known to play a major role in OA [2, 21]. To investigate if oxidative stress is an early or late event in OA and to better understand how it affects cartilage and chondrocytes, we used an antibody against oxidized type II collagen (oxPTM-CII). Anti-oxPTM-CII was developed to recognize different forms of oxidized type II collagen and was demonstrated to bind human OA and RA cartilage, but not healthy cartilage [12]. OxPTM-CII was detected in the medial tibial cartilage of the operated knees as early as 1 and 3 days after ACTL+pMx and DMM surgeries, respectively, and before any cartilage damage was visible. Until 14 days post-surgery, the staining localized in the deep zone in the pericellular and territorial matrix of large hypertrophic chondrocytes. When larger defects occurred however, oxPTM-CII extended to the interterritorial matrix through the complete depth of cartilage. The staining was absent from the lateral tibial and femoral condylar cartilage but with the progression of the disease, the staining became visible in both these compartments (day 14 for ACLT+pMx, day 28 for DMM). This is in accordance with the observations from others [9, 22] that in these OA models, lesions develop primarily in the medial tibial plateau. We also looked at the contralateral knees and found that oxPTM-CII staining was absent or weak at early time points but started to appear at later time points. This finding suggests that the disease also starts to develop in the contralateral knee, possibly because the animal changes their gait thus inducing mechanical stress in the contralateral joint [23]. This is also expected to be accelerated in the colony housing where animal are free to perform more weight-bearing activities compared to smaller cages [16, 20]. It was already demonstrated that under mechanical stress chondrocytes produce ROS [24]. It is also known that removal or displacement of the meniscus increases peak stress in the medial compartment [25] and that cartilage normally covered by the meniscus possesses decreased load-bearing capacity and less resilience to damage compared to cartilage not covered by menisci [26]. We postulate that the DMM and the ACLT+pMx surgeries produce a strong mechanical stress in the tibial cartilage that was covered by the meniscus before the surgery resulting in ROS production [2] and the subsequent appearance of oxidized type II collagen. The localization of the staining likely corresponds to the zone where mechanical stress was the highest. It is also interesting to note that on the lateral compartment where the meniscus remained, and consequently mechanical stress was lower, the staining was more diffuse and not restricted to the deep zone. A similar pattern was observed in the medial condyles. Possibly, in medial tibial cartilage, the production of ROS and the resulting oxPTM-CII arises from excessive loading while on the lateral side and the condyles the ROS production and oxPTM-CII staining might be rather due to the diffusion of ROS and other inflammatory components from the medial tibial cartilage to other joint compartments.

Because oxPTM-CII was predominantly found in the deep zone and given that ROS are known to stimulate chondrocyte hypertrophy [27, 28], we also evaluated if oxPTM-CII co-localizes with type X collagen (CX). Indeed, we observed a strong co-localization and both staining became more intense with disease progression. We also observed that 28 days after DMM surgery, CX staining extended to the complete depth of cartilage at a time when large defects were also observed, resulting in a larger area positively stained for CX. Previous studies also described an increased CX expression during OA [29, 30] and in line with our observation, a pericellular staining in the deep zone and in advanced OA also in the middle zone was reported [30]. However, to our knowledge, the present study is the first that shows an increase of CX expression early in the disease. Similarly to oxPTM-CII, CX staining was detected before any apparent cartilage damage develops. At later time points (14 days for ACLT+pMx and 28 days for DMM), staining no longer fully co-localized. The co-localization was still found in the deep zone as observed at earlier time points but staining only partially co-localized in the middle and the superficial zone. This finding demonstrates that the formation of oxPTM-CII was not restricted to hypertrophic cells. We hypothesize that chondrocytes first produce ROS, which induces hypertrophy [27, 28] and subsequently lead to CX deposition. Possibly cells from the middle zone and superficial layer are more resistant to hypertrophy [31] increasing the delay between ROS production and CX production in these zones. However, the chondrocytes in this zone might have already been pre-hypertrophic and with this in mind it would have been of interest to investigate the presence of Runx2, an early hypertrophy marker. Taken together, our results confirm the therapeutic potential of inhibiting ROS to treat OA. Indeed, it has already been demonstrated that oral administration of the anti-oxidant N-acetyl cysteine (NAC) protects against OA in the rat [32] and reduces hypertrophy in the growth plate of mice [28]. Future studies could use NAC to evaluate its impact on oxPTM-CII generation, hypertrophy, and OA initiation and progression in animal models.

Interestingly, the quantification of the medial tibial cartilage area positive for oxPTM-CII and CX revealed that in the DMM model, the CX staining spread across a larger cartilage area at day 14 already when compared with day 28 for oxPTM-CII. This may indicate that beside ROS production other mechanisms might induce cartilage hypertrophy in OA.

In addition, our results are in accordance with previous observations that the localization of oxPTM-CII is predominantly in the ipsilateral joint and that it is detected ahead of cartilage structural changes [12, 13]. In previous work, we used Cy5.5-anti-ROS CII antibody or Cy5.5-anti-ROS CII scFv injected i.v. or i.a. to detect OA changes in vivo with non-invasive imaging in DMM mice after 4 or 8 weeks. The present study demonstrates that labeled oxPTM-CII antibody or scFv could diagnose OA even earlier in the DMM model and could be used to monitor disease progression. Future longitudinal studies will need to assess the utility of anti-oxPTM-CII as a novel molecular imaging tool to both detect early onset and to longitudinally monitor OA in small animal models. In addition, because at early time points the oxPTM-CII staining was restricted to few cells in the deep zone, the anti-oxPTM-CII might need to be optimized to diffuse efficiently in cartilage and provide a high signal-to-noise ratio. If proven to be successful, anti-oxPTM-CII may be exploited for molecular imaging in parallel with future developments of MRI capabilities in human. Imaging with anti-oxPTM-CII may be interpreted in association with MRI/radiography for enhanced overall clinical management of patients, as well as improvement of outcome readouts in clinical trials.

In conclusion, oxPTM-CII and CX staining of ACLT+pMx and DMM rat knees showed that the disease starts extremely early (day 1 and 3, respectively) in the deep zone of tibial medial cartilage and that the load-bearing zone that was covered by the meniscus before surgery was affected first. Our results confirm that chondrocyte hypertrophy is an integral part of the OA pathobiology, and we propose that it might be an initiating event of the disease. In addition, because oxPTM-CII and CX staining were strictly localized in the pericellular matrix at early time points, this study also supports the hypothesis that OA is a disease of the pericellular matrix [33, 34].

## Conclusions

We propose that oxPTM-CII antibodies or oxPTM-CII scFv labeled with a fluorescent probe is a promising biomarker to detect OA initiation ahead of radiographic changes and monitor its progression.

## Supplementary Information

**Additional file 1: Table S1.** Sub-scores for the histological scoring of OA. **Figure S1.** Histological sub-scores for the medial tibial plateau in the DMM model. Rats underwent DMM surgery and were sacrificed at days 3, 5, 7,14 and 28. The ipsilateral or contralateral knees were taken for histological analysis. Slides were stained with toluidine blue and saffron du Gatinais and evaluated according to the sub-scores detailed in the Table S1. Individual data for each animal (*N* = 9–10) and the mean is shown for each timepoint as well as for the selected contralateral knees. *, ** and *** means significantly different from contralateral with *p* < 0.05, *p* < 0.01 or *p* < 0.001 respectively. **Figure S2.** Histological sub-scores for the medial tibial plateau in the ACLT+pMx model. Rats underwent ACLT+pMx surgery and were sacrificed at days 1, 3, 5, 7 and 14. The ipsilateral or contralateral knees were taken for histological analysis. Slides were stained with toluidine blue and saffron du Gatinais and evaluated according to the sub-scores detailed in the Table S1. Individual data for each animal (N = 9–10) and the mean is shown for each timepoint as well as for the selected contralateral knees. *, **, *** and **** means significantly different from contralateral with p < 0.05, 0.01, 0.001, and 0.0001 respectively. The sub-score chondroosteophyte is not shown because it was equal to 0 in all groups. **Figure S3.** Staining and total histological score for the lateral tibial plateaus in the ACLT+pMx and DMM models. **A.** At various timepoints, the knees were taken for histological analysis and stained with toluidine blue and saffron du Gatinais or for type X collagen (blue, CX) and oxPTM type II collagen (brown, oxPTM-CII). Staining for the lateral tibial plateau for different timepoints and scores obtained for the medial plateau (in brackets) are shown. **B.** The total histological score for the lateral tibial plateau was determined according to the sub-scores detailed in the Table S1. Individual data for each animal (*N* = 9–10) and the mean is shown for each timepoint and selected contralateral knees. * means significantly different from contralateral with *p* < 0.05. **Figure S4.** Staining of the contralateral tibial plateaus in the ACLT+pMx and DMM models. Representative pictures obtained with the toluidine blue and saffron du Gatinais staining, type X collagen (in blue, CX) and oxPTM-type II collagen (in brown, oxPTM-CII) single or double immunostainings are shown.

## Data Availability

All data generated during this study are included in this published article and in its supplementary information files or are available from the corresponding author on reasonable request.

## Abbreviations

OA: Osteoarthritis
CII: Collagen type II
oxPTM-CII: Oxidative post-translationally modified collagen type II
CX: Collagen type X
ACLT+pMx: Anterior cruciate ligament transection with partial meniscectomy
DMM: Destabilization of the medial meniscus
ROS: Reactive oxidants
