# Supplementary material for: Early detection of osteoarthritis in the rat with an antibody specific to type II collagen modified by reactive oxygen species
Source: Arthritis Res Ther. 2021 Apr 14;23:113. doi: 10.1186/s13075-021-02502-1 (PMC8045329; doi:10.1186/s13075-021-02502-1)

**Table S1: Sub-scores for the histological scoring of OA**

| Parameters | Grade | Description |
| --- | --- | --- |
| Cartilage structure | 0  1  2  3  4  5  6  7  8 | Normal, smooth, uninterrupted surface  Mild surface irregularities (undulations)  Irregular surface, 1-3 superficial clefts (fissures)  >3 fissures and/or loss of cartilage in the superficial zone  1-3 fissures extending into the middle zone  >3 fissures and/or loss of cartilage extending into the middle zone  1-3 fissures extending into the deep zone  >3 fissures extending into the deep zone and/or loss of cartilage to deep zone  Fissures or loss of cartilage extending to the zone of calcified cartilage |
| Matrix staning | 0  1  2  3  4  5  6 | Uniform throughout articular cartilage  Decreased in superficial zone only and for ˂half the length of the condyle or plateau  Decreased in superficial zone for half the length or greater of the condyle or plateau  Decreased in superficial and middle zones for ˂half the length of the condyle or plateau  Decreased in superficial and middle zones for half the length or greater of the condyle or plateau  Decreased in all 3 zones for ˂half the length of the condyle or plateau  Decreased in all 3 zones for half the length or greater of the condyle or plateau |
| Cellularity | 0  1  2  3  4 | Normal (1-2 cells/lacuna)  Slight hypocellularity  Regions of hypocellularity and/ or clustering  Strong hypocellularity  No cells (or cartilage loss down to tidemark) |
| Tidemark | 0  1  2 | Intact/single tidemark  Duplication of tidemark  Interruption or loss of tidemark |
| Subchondral bone | 0  1  2  3  4 | Normal  25% thickening, in particular just below the cartilage  26-50% thickening of the subchondral bone  51-75% thickening of the subchondral bone  >75 thickening of the subchondral bone |
| Chondroosteophyte | 0  1  2  3 | No osteophyte visible  Small osteophyte  Medium-sized osteophyte  Large osteophyte |

**Figure S1: Histological sub-scores for the medial tibial plateau in the DMM model.** Rats underwent DMM surgery and were sacrificed at days 3, 5, 7,14 and 28. The ipsilateral or contralateral knees were taken for histological analysis. Slides were stained with toluidine blue and saffron du Gatinais and evaluated according to the sub-scores detailed in the Table S1. Individual data for each animal (N=9-10) and the mean is shown for each timepoint as well as for the selected contralateral knees. *, ** and *** means significantly different from contralateral with p<0.05, p<0.01 or p<0.001 respectively.


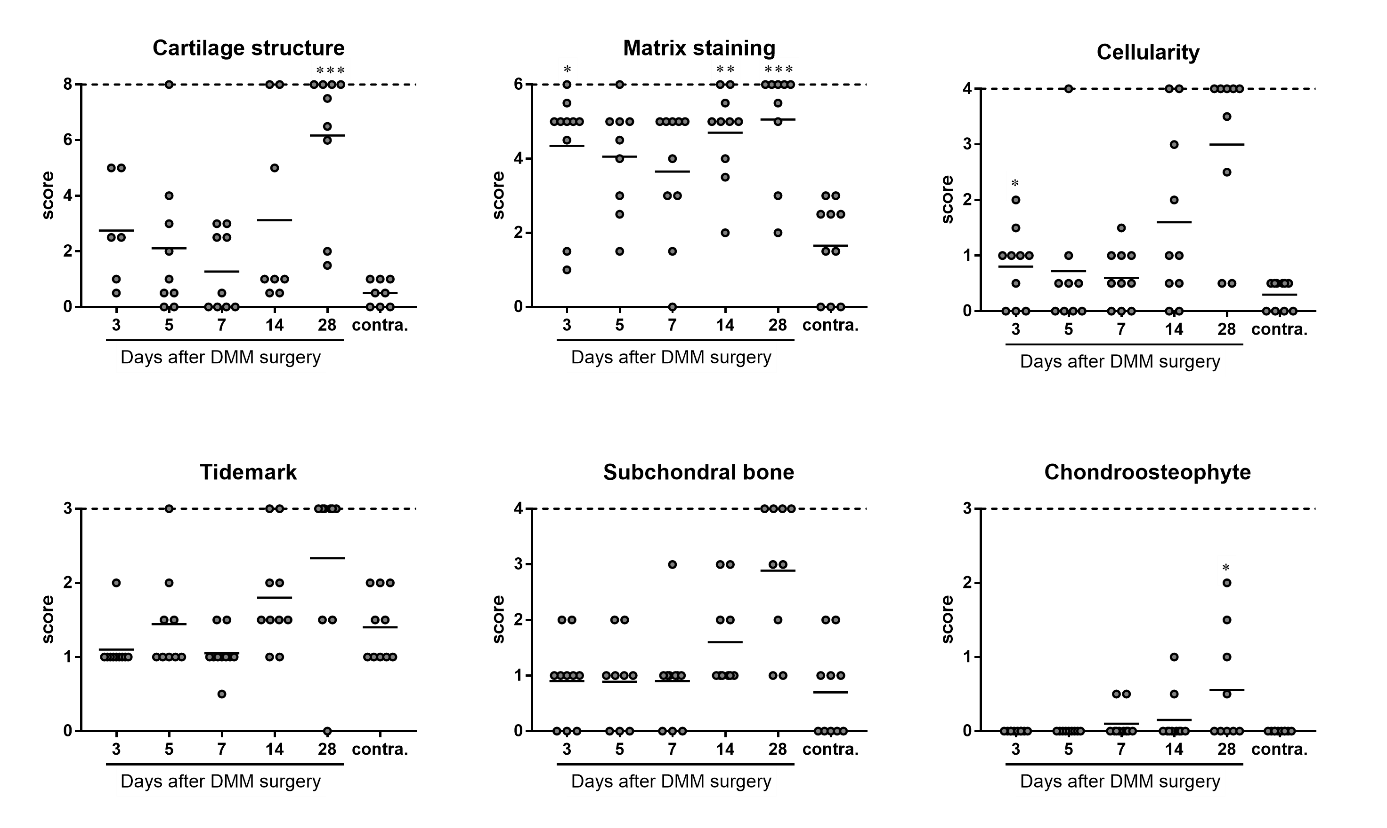


**Figure S2: Histological sub-scores for the medial tibial plateau in the ACLT+pMx model.** Rats underwent ACLT+pMx surgery and were sacrificed at days 1, 3, 5, 7 and 14. The ipsilateral or contralateral knees were taken for histological analysis. Slides were stained with toluidine blue and saffron du Gatinais and evaluated according to the sub-scores detailed in the Table S1. Individual data for each animal (N=9-10) and the mean is shown for each timepoint as well as for the selected contralateral knees. *, **, *** and **** means significantly different from contralateral with p<0.05, 0.01, 0.001, and 0.0001 respectively. The sub-score chondroosteophyte is not shown because it was equal to 0 in all groups.


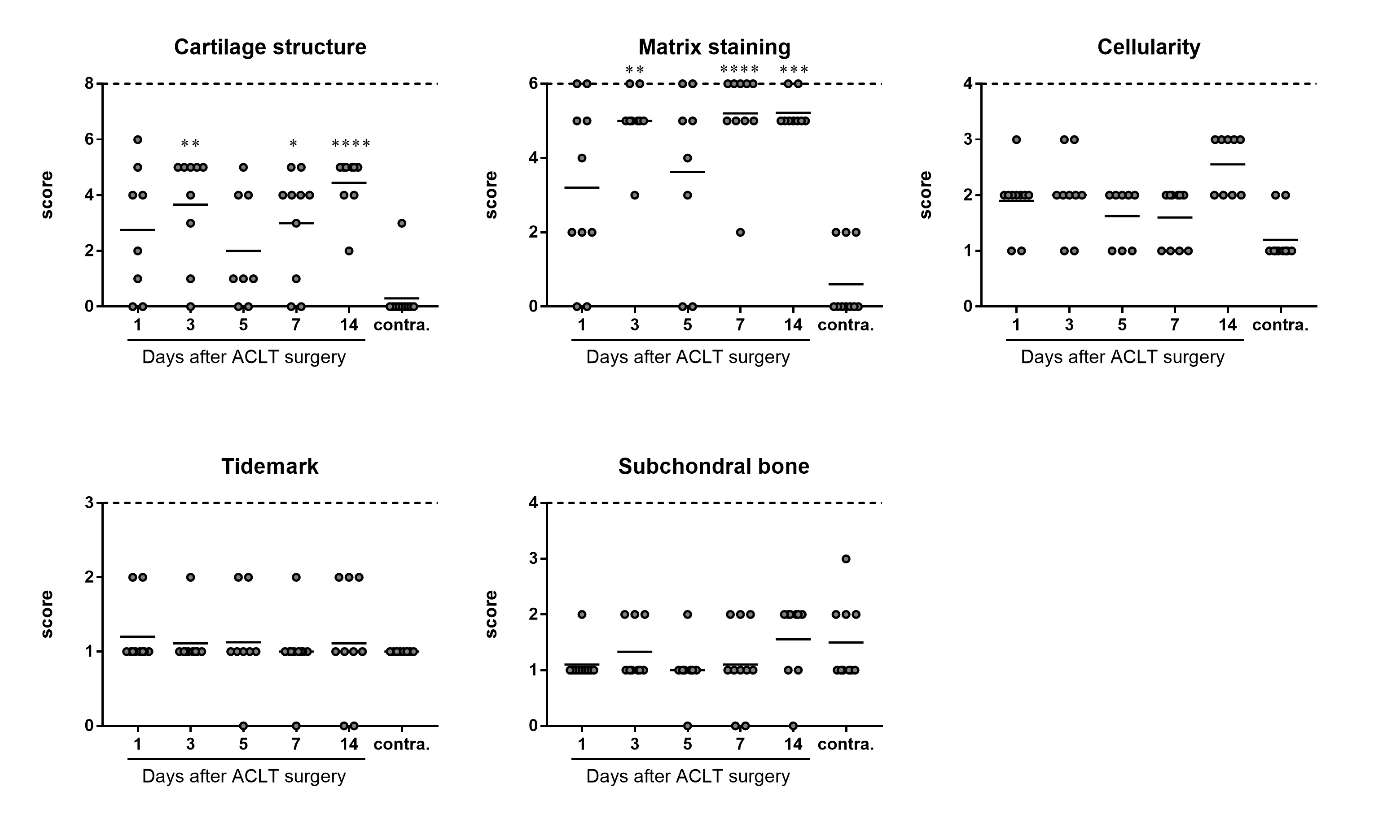


**Figure S3: Staining and total histological score for the lateral tibial plateaus in the ACLT+pMx and DMM models. A.** At various timepoints, the knees were taken for histological analysis and stained with toluidine blue and saffron du Gatinais or for type X collagen (blue, CX) and oxPTM type II collagen (brown, oxPTM-CII). Staining for the lateral tibial plateau for different timepoints and scores obtained for the medial plateau (in brackets) are shown. **B.** The total histological score for the lateral tibial plateau was determined according to the sub-scores detailed in the Table S1. Individual data for each animal (N=9-10) and the mean is shown for each timepoint and selected contralateral knees. * means significantly different from contralateral with p<0.05.


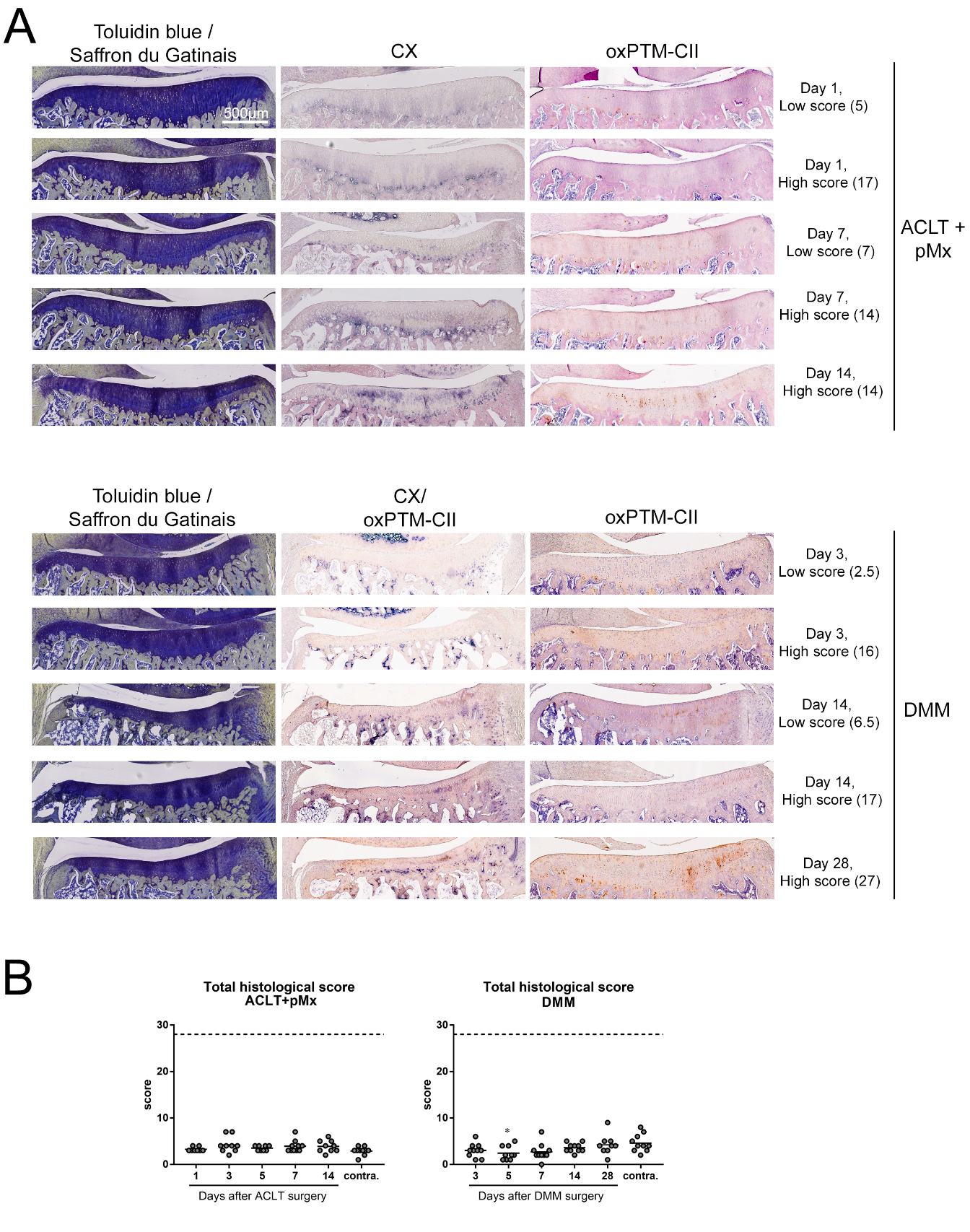


**Figure S4: Staining of the contralateral tibial plateaus in the ACLT+pMx and DMM models.** Representative pictures obtained with the toluidine blue and saffron du Gatinais staining, type X collagen (in blue, CX) and oxPTM-type II collagen (in brown, oxPTM-CII) single or double immunostainings are shown.


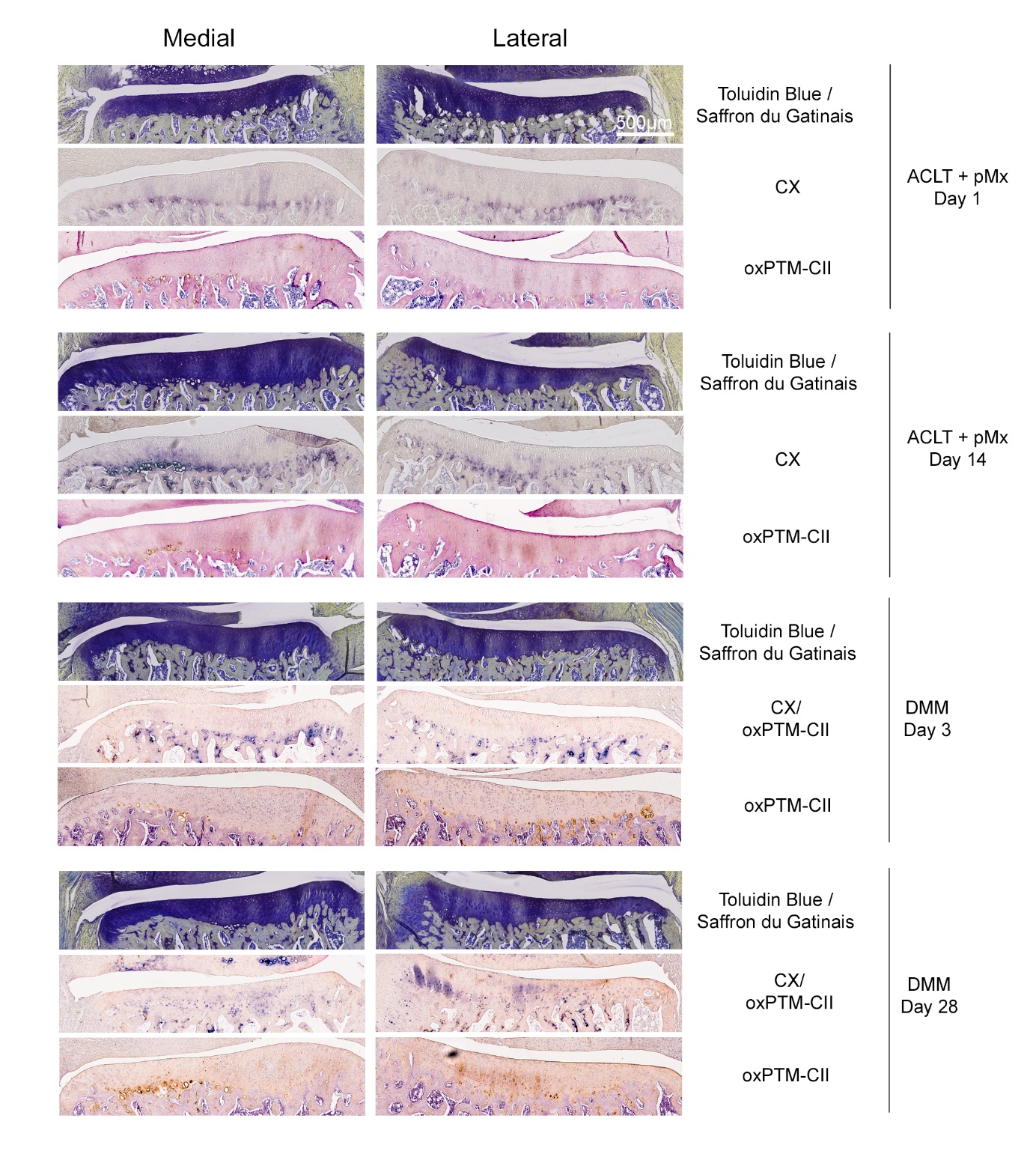

Supplement: Supplementary file 1 — Additional file 1: Table S1. Sub-scores for the histological scoring of OA. Figure S1. Histological sub-scores for the medial tibial plateau in the DMM model. Rats underwent DMM surgery and were sacrificed at days 3, 5, 7,14 and 28. The ipsilateral or contralateral knees were taken for histological analysis. Slides were stained with toluidine blue and saffron du Gatinais and evaluated according to the sub-scores detailed in the Table S1. Individual data for each animal (N = 9–10) and the mean is shown for each timepoint as well as for the selected contralateral knees. *, ** and *** means significantly different from contralateral with p < 0.05, p < 0.01 or p < 0.001 respectively. Figure S2. Histological sub-scores for the medial tibial plateau in the ACLT+pMx model. Rats underwent ACLT+pMx surgery and were sacrificed at days 1, 3, 5, 7 and 14. The ipsilateral or contralateral knees were taken for histological analysis. Slides were stained with toluidine blue and saffron du Gatinais and evaluated according to the sub-scores detailed in the Table S1. Individual data for each animal (N = 9–10) and the mean is shown for each timepoint as well as for the selected contralateral knees. *, **, *** and **** means significantly different from contralateral with p < 0.05, 0.01, 0.001, and 0.0001 respectively. The sub-score chondroosteophyte is not shown because it was equal to 0 in all groups. Figure S3. Staining and total histological score for the lateral tibial plateaus in the ACLT+pMx and DMM models. A. At various timepoints, the knees were taken for histological analysis and stained with toluidine blue and saffron du Gatinais or for type X collagen (blue, CX) and oxPTM type II collagen (brown, oxPTM-CII). Staining for the lateral tibial plateau for different timepoints and scores obtained for the medial plateau (in brackets) are shown. B. The total histological score for the lateral tibial plateau was determined according to the sub-scores detailed in the Table S1. Indivi [file 13075_2021_2502_MOESM1_ESM.docx]
